# Fungal Carbonyl Sulfide Hydrolase of *Trichoderma harzianum* Strain THIF08 and Its Relationship with Clade D β-Carbonic Anhydrases

**DOI:** 10.1264/jsme2.ME20058

**Published:** 2021-05-22

**Authors:** Yoshihito Masaki, Ryuka Iizuka, Hiromi Kato, Yuka Kojima, Takahiro Ogawa, Makoto Yoshida, Yasuhiko Matsushita, Yoko Katayama

**Affiliations:** 1 Graduate School of Agriculture, Tokyo University of Agriculture and Technology, 3–5–8 Saiwai-cho, Fuchu, Tokyo 183–8509, Japan; 2 Graduate School of Life Sciences, Tohoku University, 2–1–1 Katahira, Sendai 980–8577, Japan; 3 Gene Research Center, Tokyo University of Agriculture and Technology, 3–5–8 Saiwai-cho, Fuchu, Tokyo 183–8509, Japan; 4 Independent Administrative Institution, Tokyo National Research Institute for Cultural Properties, 13–43 Ueno Park, Taito-ku, Tokyo 110–8713, Japan

**Keywords:** clade D β-carbonic anhydrase, carbonyl sulfide, COS hydrolase, *Trichoderma harzianum*

## Abstract

Carbonyl sulfide (COS) is the most abundant and long-lived sulfur-containing gas in the atmosphere. Soil is the main sink of COS in the atmosphere and uptake is dominated by soil microorganisms; however, biochemical research has not yet been conducted on fungal COS degradation. COS hydrolase (COSase) was purified from *Trichoderma harzianum* strain THIF08, which degrades COS at concentrations higher than 10,000 parts per million by volume from atmospheric concentrations, and its gene *cos* (492 bp) was cloned. The recombinant protein purified from *Escherichia coli* expressing the *cos* gene converted COS to H_2_S. The deduced amino acid sequence of COSase (163 amino acids) was assigned to clade D in the phylogenetic tree of the β-carbonic anhydrase (β-CA) family, to which prokaryotic COSase and its structurally related enzymes belong. However, the COSase of strain THIF08 differed from the previously known prokaryotic COSase and its related enzymes due to its low reactivity to CO_2_ and inability to hydrolyze CS_2_. Sequence comparisons of the active site amino acids of clade D β-CA family enzymes suggested that various *Ascomycota*, particularly *Sordariomycetes* and *Eurotiomycetes*, possess similar enzymes to the COSase of strain THIF08 with >80% identity. These fungal COSase were phylogenetically distant to prokaryotic clade D β-CA family enzymes. These results suggest that various ascomycetes containing COSase contribute to the uptake of COS by soil.

Carbonyl sulfide (COS; O=C=S; carbon oxysulfide) is the most abundant sulfur gas in the troposphere, with an average reported mixing ratio of approximately 500 parts per trillion by volume (pptv). Measurements of atmospheric COS fluxes in terrestrial environments are considered to be important because they may be used as a proxy for estimating gross primary production (GPP) due to the clear relationship between the uptakes of COS and CO_2_ by vegetation (Goldan *et al.*, 1988; Protoschill-Krebs and Kesselmeier, 1992; Kesselmeier and Merk, 1993; Sandoval-Soto *et al.*, 2005; Stimler *et al.*, 2010; Berry *et al.*, 2013; Campbell *et al.*, 2017; Whelan *et al.*, 2018). Therefore, a detailed understanding of the role of soil microorganisms that produce or degrade COS is important (Masaki *et al.*, 2016).

COS uptake in terrestrial ecosystems is mainly performed by vegetation and soil, which account for approximately 70 to 90% of the COS global sink (Watts, 2000; Kettle *et al.*, 2002; Montzka *et al.*, 2007; Berry *et al.*, 2013; Launois *et al.*, 2015; Whelan *et al.*, 2018). Plants take up COS by photosynthetic enzymes including carbonic anhydrase (CA, EC 4.2.1.1) and ribulose-1,5-bisphosphate carboxylase/oxygenase (RuBisCO, EC 4.1.1.39) (Lorimer and Pierce, 1989; Ogée *et al.*, 2016) (Table S1). CO_2_ is a substrate for CA (CO_2_+H_2_O ⇄ HCO_3_^–^+H^+^), and since COS and CO_2_ are structurally similar, COS is also hydrolyzed by CA to produce H_2_S (COS+H_2_O → CO_2_+H_2_S). CA and RuBisCO are both essential for CO_2_ fixation in plants, abundant in the biosphere, and thus, make the greatest contribution to the consumption of COS in the troposphere (Ellis, 1979; Badger and Price, 1994). In addition to CO_2_ fixation, CA has diverse functions and is widely distributed in three major families (α-, β-, and γ-CAs). There are four phylogenetically distinct clades within the β-CA family (clades A to D) (Smith *et al.*, 1999; Smith and Ferry, 2000).

COS-degrading enzymes involved in chemolithotrophic energy production have been discovered in bacteria (COS hydrolase, COSase, EC 3.13.1.7) and archaea (carbon disulfide [CS_2_] hydrolase, EC 3.13.1.5) (Table S1). COSase was initially purified from *Thiobacillus thioparus* strain THI115, a chemolithoautotrophic sulfur-oxidizing proteobacterium that turns COS into H_2_S and uses as the sole electron donor for energy metabolism. Although CO_2_ hydration activity is markedly weaker than those of β-CA family members, a comparison of amino acid sequences revealed that the COSase of strain THI115 belongs to the clade D cluster of the phylogenetic tree in the β-CA family (Ogawa *et al.*, 2013). Not only chemolithotrophic sulfur bacteria, but also various bacteria belonging to the phylum *Actinobacteria* were found to have the amino acid sequences of clade D β-CA family enzymes, suggesting the importance of *Actinobacteria* in the degradation of COS in soil environments (Ogawa *et al.*, 2016; Kato *et al.*, 2020).

Previous studies on soil treated with autoclave sterilization or a CA inhibitor demonstrated that soil COS consumption is a biological process (Saito *et al.*, 2002) and involves microbial CA (Kesselmeier *et al.*, 1999). Soil microorganisms take up high concentrations of COS without the need for acclimation (Saito *et al.*, 2002). COS-degrading bacteria belonging to the genera *Mycobacterium*, *Williamsia*, and *Cupriavidus* and fungi belonging to the *Trichoderma*, *Beauveria* and *Scytalidium* were isolated from forest soils exhibiting COS-degrading activity (Kato *et al.*, 2008, Masaki *et al.* 2016). In addition, COS-degrading bacteria have been detected in a wide range of *Actinomycetes* (Kusumi *et al.*, 2011; Ogawa *et al.*, 2016) and in *Acidithiobacillus* (Smeulders *et al.*, 2013). The enzyme involved and the distribution of COS-degradation ability in fungi have yet to be studied.

In the present study, we describe the identification, gene cloning, and characterization of the COSase of *Trichoderma* sp. strain THIF08. Comparisons of the amino acid sequence revealed that the COSase of strain THIF08 is a novel β-CA family enzyme that belongs to clade D in this family.

## Materials and Methods

### Organism and culture conditions

*Trichoderma* sp. strain THIF08 was isolated from forest soil and was maintained on potato dextrose agar (PDA, Nihon Pharmaceutical) as previously described (Masaki *et al.*, 2016). The morphological characteristics of strain THIF08 were observed using mycelia that formed on PDA plates. To purify COSase, strain THIF08 was cultivated in potato dextrose liquid medium, pH 5.9 (Potato dextrose broth; Difco, Becton Dickinson) with reciprocal shaking at 120‍ ‍rpm in air (approximately 500 pptv COS) for 4 d. Cells were cultivated at 30°C in the dark.

### Phylogenetic identification of *Trichoderma* sp. strain THIF08

Genomic DNA was extracted from the mycelia of strain THIF08 using an Isoplant DNA extraction kit (Nippon Gene). The ITS1-5.8S-ITS2 ribosomal RNA (ITS) region was amplified by PCR with the ITS5_primer and ITS4_primer (Table S2) (White *et al.*, 1990). The translation elongation factor 1 alpha (tef1α) region was amplified by PCR with the TEF1_primer and TEF728_primer (Table S2) (Dees and Ghiorse, 2001). PCR products were sequenced by Macrogen Japan. The sequences obtained were compared with data in DDBJ/EMBL/GenBank using the BLAST program to identify phylogenetic neighbors.

### Activity stain for the detection of COSase

Cells of strain THIF08 were collected by centrifugation at 15,500×*g* at 4°C for 20‍ ‍min, rinsed with 50‍ ‍mM Tris-HCl, pH 9.0, and then stored at –80°C. Cells (5‍ ‍g as wet weight defrosted) were re-suspended in 15‍ ‍mL of 50‍ ‍mM Tris-HCl, pH 9.0, and disrupted using a three-dimensional bead impact homogenizer (Shake Master Auto; Bio-Medical Science) at 4°C in a 25-mL volume with zirconia beads (0.6‍ ‍mm in diameter) (Bio-Medical Science). Cell debris was removed by centrifugation at 20,000×*g* at 4°C for 20‍ ‍min, and the cell-free extract was then filtered through glass microfiber filter paper with a pore size of 1.2‍ ‍μm (grade GF/C; Whatman, GE Healthcare Life Sciences). A 30-μL aliquot of the filtrate was subjected to native polyacrylamide gel electrophoresis (PAGE) on an 8% gel (Davis, 1964). After electrophoresis, the gel was soaked in 500‍ ‍mL of 50‍ ‍mM Tris-HCl, pH 9.0 containing 78‍ ‍mg of bismuth trichloride in a sealable plastic container. The enzyme reaction was started by bubbling COS gas (100,000 parts per million by volume [ppmv] with N_2_ as the balance gas, Nissan Tanaka Corporation), and the gel was incubated at 30°C for 2 h in the dark. Bismuth in the solution reacted with sulfide produced by COS hydrolysis and formed a black precipitate at locations on the polyacrylamide gel at which the enzyme was present (Claesson *et al.*, 1990; Smeulders *et al.*, 2011). The area visualized by the black precipitate (molecular mass: approximately 60‍ ‍kDa, 1×1‍ ‍cm) was excised from the gel and placed in a dialysis cellulose tube (nominal molecular mass cut-off value: 10‍ ‍kDa) with 5‍ ‍mL of 50‍ ‍mM Tris-HCl, pH 9.0. The protein was then electrically eluted from the gel. The protein solution was concentrated approximately 10-fold by an ultrafiltration cell fit with a YM-10 membrane (Millipore, Merck). A 30-μL aliquot of the resulting solution was precipitated with acetone, dissolved in 50‍ ‍mM Tris-HCl, pH 9.0, and then subjected to sodium dodecyl sulfate (SDS)-PAGE on a 12.5% gel.

### Purification of COSase

The cell-free extract filtered as described above (10‍ ‍mL) was loaded onto a column (1×5‍ ‍cm) packed with a weak anion exchange chromatography resin (DEAE-Sepharose; Sigma-Aldrich, Merck), equilibrated in 50‍ ‍mM Tris-HCl, pH 9.0. Proteins were eluted from the column with a KCl gradient from 0 to 0.6 M in 50‍ ‍mM Tris-HCl, pH 9.0, at a flow rate of 1‍ ‍mL min^–1^. Active fractions showing H_2_S production in the presence of COS monitored using a gas chromatograph-flame photometric detector (GC-FPD) (GC-14B; Shimadzu) were pooled and then incubated at 4°C for 2 h after the addition of ammonium sulfate (30% final concentration). After centrifugation at 20,000×*g* at 4°C for 10‍ ‍min, the supernatant was loaded onto a column (1×5‍ ‍cm) packed with a strong anion exchange chromatography resin (Toyopearl SuperQ; Tosoh), equilibrated in 50‍ ‍mM Tris-HCl, pH 9.0, containing 30% ammonium sulfate. Unbound proteins were obtained by washing the column with 50‍ ‍mM Tris-HCl, pH 9.0, at a flow rate of 1‍ ‍mL min^–1^. Fractions exhibiting COSase activity were loaded onto a strong anion exchange chromatography column (1‍ ‍mL) (HiTrap Q HP; GE Healthcare Life Sciences) equilibrated in 50‍ ‍mM Tris-HCl, pH 9.0. Proteins were eluted from the column with a NaCl gradient from 0 to 0.6 M in 50‍ ‍mM Tris-HCl, pH 9.0, at a flow rate of 1‍ ‍mL min^–1^. Active fractions were then loaded onto a weak anion exchange chromatography column (1‍ ‍mL) (HiTrap DEAE FF; GE Healthcare Life Sciences) equilibrated in 50‍ ‍mM Tris-HCl, pH 9.0. Proteins were eluted with 0.6 M NaCl in 50‍ ‍mM Tris-HCl, pH 9.0, at a flow rate of 1‍ ‍mL min^–1^. Fractions exhibiting COSase activity were pooled and subjected to SDS-PAGE.

### Measurement of COSase activity

COSase activity was measured in a brown glass vial (4.9‍ ‍mL) sealed with butyl rubber stopper containing 200‍ ‍μL of the reaction mixture of enzyme solution and 50‍ ‍mM Tris-HCl buffer, pH 9.0, and then incubated at 30°C. The reaction was started by adding COS gas after removing an equal volume of headspace gas to make the final COS concentration approximately 1,000–2,000 ppmv. During the incubation, the headspace gas (50‍ ‍μL) was sampled at appropriate intervals, and COS and H_2_S were measured using GC-FPD as previously described (Kato *et al.*, 2008). All enzyme assays were conducted in duplicate with 50‍ ‍mM Tris-HCl buffer, pH 9.0, as a negative control.

### CS_2_ hydrolysis activity

The procedure employed to measure CS_2_ hydrolysis activity was essentially the same as that for COSase activity, except that CS_2_ (Wako Pure Chemical Industries) was used instead of COS. The initial CS_2_ concentration in the reaction mixture was 460‍ ‍μM or 80‍ ‍mM. The amount of H_2_S in the headspace gas was measured by GC-FPD.

### CA activity

The protocol for the measurement of CA activity was based on the description by Wilbur and Anderson (Wilbur and Anderson, 1948) with some modifications. CA activity was calculated using Wilbur–Anderson units (WAU) mg^–1^ protein. The definition for WAU is (T_blank_–T_req_)/T_req_ where T_blank_ and T_req_ are the times required for the pH of the reaction mixture to change from pH 8.3 to 7.0 with or without an enzyme. Two milliliters of CO_2_-saturated water was added to glass vials containing 3‍ ‍mL of ice-cold 20‍ ‍mM Tris-sulfate, pH 8.3. After the pH of the reaction mixture had reached approximately pH 8.4–8.5, the reaction was started by the addition of 50‍ ‍μL of ice-cold enzyme solution in 50‍ ‍mM Tris-HCl, pH 8.5 (at 25°C). The enzyme solution contained 5‍ ‍μg of GST-fused COSase (the amount equivalent to 2‍ ‍μg protein of the region of interest) or 1‍ ‍μg of bovine CA Isozyme II (Sigma-Aldrich). pH changes during the assay were recorded with a pH meter (F-72; HORIBA) and a probe (9618S-10D; HORIBA) with gentle stirring. The experiment was performed at a temperature range between 0 and 3°C. Samples were prepared in triplicate.

### Effects of CO_2_

CO_2_ gas was added to the reaction vial containing the enzyme solution and buffer (Nissan Tanaka Corporation) after removing an equal volume of headspace gas to reach a final CO_2_ concentration of 100, 1,000, or 10,000 ppmv. Headspace air was replaced by nitrogen gas (Taiyo Nissan) prior to the experiment.

### Quantification of protein

The enzyme preparation was mixed with an equal volume of buffer containing 2.1% SDS, 5% mercaptoethanol, 65.8‍ ‍mM Tris-HCl, pH 6.8, and heated at 100°C for 5‍ ‍min. After centrifugation at 16,000×*g* at 4°C for 1‍ ‍min, the resultant supernatant was subjected to ultrafiltration using Vivaspin 500 3,000K MWCO PES (Sartorius) to decrease the buffer concentration to 10^–2^. Quantification of the protein solution was calculated by absorbance at 280‍ ‍nm with the molar absorption coefficient (40,920 M^–1^ cm^–1^ for GST and 44,880 M^–1^ cm^–1^ for GST-fused COSase) calculated according to the formula by Gill and Hippel (Gill and Hippel, 1989).

### N-terminal protein sequencing

The purified enzyme was subjected to SDS-PAGE and then electroblotted onto a polyvinylidene difluoride membrane. The enzyme was visualized with Coomassie brilliant blue (CBB) staining and then sent to the Institute for Protein Research (Osaka University, Osaka, Japan) to elucidate the N-terminal amino acid sequence using Edman degradation.

### Cloning of genes encoding COSase and glucose-6-phosphate isomerase (GPI)

Genomic DNA was isolated from *Trichoderma* sp. strain THIF08 using the Isoplant DNA extraction kit (Nippon Gene) according to the manufacturer’s instructions. PCR for cloning was performed using Phusion High-Fidelity DNA polymerase (Finnzymes).

### Production of COSase in *Escherichia coli*

To identify COSase activity, *E. coli* XL10-Gold (Agilent) was transformed with the pGEX-6P-1 (GE Healthcare Life Sciences), pGEX-COS, or pGEX-GPI plasmid and grown to the mid-exponential phase in 100‍ ‍mL of LB medium containing ampicillin (0.1‍ ‍mg mL^–1^). Isopropyl β-D-1-thiogalactopyranoside (IPTG) was added to the culture at a final concentration of 0.2‍ ‍mM to induce the production of glutathione *S*-transferase (GST), GST-fused COSase, or GST-fused GPI. Cells were harvested by centrifugation at 3,000×*g* at 4°C for 10‍ ‍min, washed with 50‍ ‍mM Tris-HCl buffer, pH 9.0, and suspended in the same buffer. Cells were disrupted using glass beads (0.3‍ ‍mm in diameter) with the Mini Bead Beater (WakenBtech), and centrifuged at 20,000×*g* at 4°C for 20‍ ‍min. The supernatant was mixed with glutathione-conjugated magnetic beads (MagneGST particles; Promega) to capture GST or the GST-fused proteins of interest. The magnetic beads were washed with PreScission buffer (GE Healthcare Life Sciences; 50‍ ‍mM Tris-HCl, 150‍ ‍mM NaCl, 1‍ ‍mM ethylenediaminetetraacetic acid, and 1‍ ‍mM dithiothreitol, pH 7.0). To cleave GST from the fusion proteins, 1‍ ‍μL (2 units) of PreScission Protease (GE Healthcare Life Sciences) was added to the bead suspension and incubated on a rotating wheel at 4°C overnight. The resulting solution containing COSase, GPI, or the beads capturing GST was used to measure COSase activity.

In experiments on competitive inhibition by CO_2_, CS_2_ hydrolysis activity, and CA activity, *E. coli* BL21 (DE3) (BioDynamics Laboratory) was transformed with the pGEX-6P-1 or pGEX-COS plasmid and grown to the mid-exponential phase in 100‍ ‍mL of LB medium containing ampicillin (0.1‍ ‍mg mL^–1^). IPTG was added to the culture at a final concentration of 0.1‍ ‍mM to induce the production of GST and GST-fused COSase. Cells were harvested by centrifugation at 10,000×*g* at 4°C for 5‍ ‍min, washed with 500‍ ‍mM Tris-HCl, pH 8.0, and suspended in 5‍ ‍mL of the same buffer. Cells were disrupted by sonication and centrifuged at 10,000×*g* at 4°C for 5‍ ‍min. The resultant supernatant was mixed with Glutathione Sepharose^TM^ 4B (GE Healthcare) to capture GST or GST-fused COSase. Beads (1.2‍ ‍mL) were washed with 50‍ ‍mM Tris-HCl, pH 8.0 and resuspended in 4.8‍ ‍mL of 50‍ ‍mM Tris-HCl, pH 8.5. The resulting solution containing Glutathione Sepharose^TM^ 4B beads capturing GST-fused COSase or GST was used.

### Phylogeny of *Trichoderma* sp. strain THIF08 COSase in β-CA family enzymes

The phylogeny of COSase was inferred using the neighbor-joining method (Saitou and Nei, 1987). Evolutionary distances were computed using the Poisson correction method (Zuckenrkandl and Pauling, 1965) with the number of amino acid substitutions per site. Evolutionary analyses were conducted using Molecular Evolutionary Genetics Analysis software X (MEGA X) (v.10.0.5) (Kumar *et al.*, 2018).

### Sequence data

The nucleotide sequences of genomic DNA encoding COSase, GPI, the ITS region, and tef1α region of strain THIF08 were submitted to DDBJ under the accession numbers LC499780, LC501463, LC600319, and LC600467, respectively.

## Results

### Identification of *Trichoderma* sp. strain THIF08

*Trichoderma* sp. strain THIF08 was found during the screening of fungi exhibiting COS-degrading activity from soil isolates (Masaki *et al.*, 2016). Many fungal isolates with COS-degrading activity belonged to the genus *Trichoderma*, and strain THIF08 exhibited the strongest activity against degradation over a wide range of COS at concentrations higher than 10,000 ppmv from atmospheric concentrations (approximately 500 pptv); therefore, strain THIF08 was used for further study.

The mycelia of strain THIF08 were white aerial hyphae that were covered by yellow-green conidia with the progression of the culture and appeared to be granular or powdery due to dense conidiation. Yellow to brown pigments were distributed throughout the agar medium. Conidiophores had primary branches at regular intervals that were in pairs or spirals consisting of 3–4 branches. Phialides were flask- to cone-shaped, and 2–4 phialides mostly arose in verticils (Fig. S1). These characteristics were consistent those of fungi in the genus *Trichoderma* (Gams and Bissett, 1998).

The sequences of the ITS and tef1α regions of strain THIF08 showed high homology with the ITS regions of NBRC 31294 (JN943372.1, 100%) of *Hypocrea nigricans* and FZ1302 (HQ259308.1, 100%) of *Hypocrea lixii* (Teleomorph of *Trichoderma harzianum*), and with the tef1α regions of strain TAMA0431 (AB856677.1, 100%) and DUCC003 (HQ602998.1, 100%) of *T. harzianum*, respectively. In the strain catalog of NBRC, NBRC 31294 is listed under the name *Hypocrea nigricans*, which is the synonym for *H. lixii* (Chaverri and Samuels, 2002). Based on morphological and DNA sequence information, strain THIF08 was identified as *T. harzianum*.

### Detection of COSase by activity staining

**Fig. 1.**
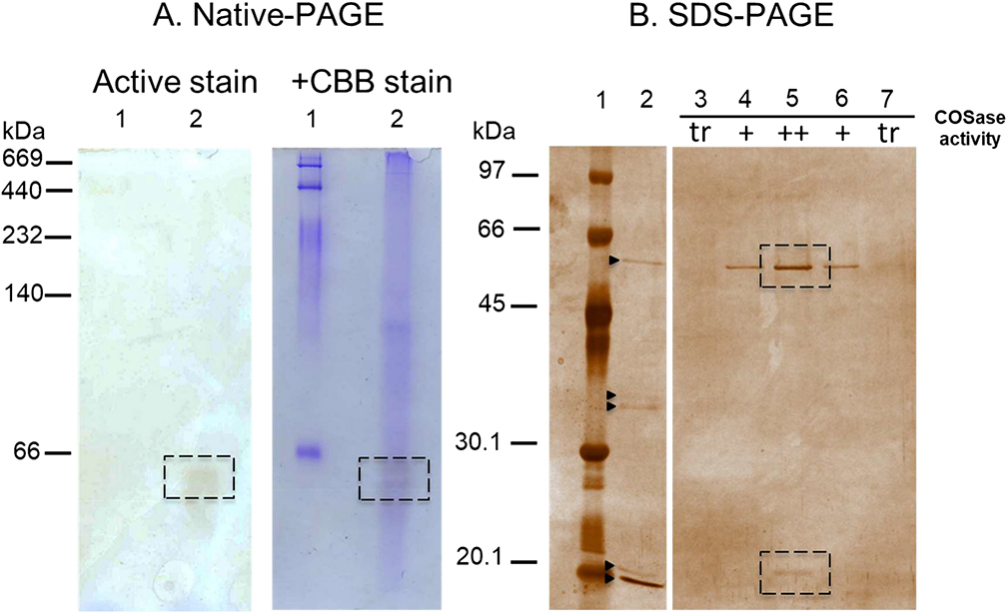
Detection of the COSase activity of *Trichoderma harzianum* strain THIF08. (A) Native-PAGE analysis of the cell-free extract from *Trichoderma harzianum* strain THIF08 exhibiting COSase activity. Active stain, Activity staining of the enzyme; +CBB stain, double staining by CBB after activity staining. Lane 1, molecular mass marker proteins; lane 2, cell-free extract (equivalent to the original cells of 20‍ ‍mg in wet wt). Dashed squares indicate the areas of the gel at which the sulfide precipitate was detected by enzyme activity staining (approximately 60‍ ‍kDa). (B) SDS-PAGE analysis of the protein solution extracted from *Trichoderma harzianum* strain THIF08. Lane 1, molecular mass marker proteins; lane 2, electrically eluted 60-kDa protein after Native-PAGE (A, lane 2); lanes 3–7, active fractions exhibiting COSase activity after Hi trap DEAE FF. One sample lane was omitted between lanes 2 and 3 in the photograph. Abbreviations: tr, trace activity; +, low activity; ++, high activity. Proteins in the gel were detected by silver staining. An electrically eluted 60-kDa protein band involved five proteins (lane 2, black triangle), in which only two bands, 20 and 60‍ ‍kDa, were consistent with the proteins in the active fraction (lane 5, dashed square).

The cell-free extract prepared from the hyphae of strain THIF08 grown on PDA medium degraded 1,700 ppmv of COS and produced H_2_S (Fig. S2). H_2_S is a reaction product of COS hydrolysis by the COSase of *T. thioparus* strain THI115 (COS+H_2_O → H_2_S+CO_2_) (Ogawa *et al.*, 2013). The heat treatment of the strain THIF08 cell-free extract at 80°C for 60‍ ‍min abolished both COS degradation and H_2_S production (Fig. S2), indicating that H_2_S was produced by the enzymatic reaction that was putatively responsible for COS degradation. Activity staining of the cell-free extract after Native-PAGE produced a single protein band with a molecular mass of ~60‍ ‍kDa (Fig. 1A). In SDS-PAGE, the excised 60-kDa band was separated into five protein bands (Fig. 1B, lane 2). Of these proteins, only two protein bands with molecular masses of 20 and 60‍ ‍kDa, respectively, were consistent with COSase activity (Fig. 1B, lanes 3–7).

### N-terminal sequencing of COSase

The partial N-terminal amino acid sequences of the 20-kDa and 60-kDa proteins were TVASEFEVAN and APANTLPAXSE (X indicates an unidentified amino acid residue), respectively. A search of the National Center for Biotechnology Information (NCBI, Bethesda, MD, USA) database using the BLASTp program revealed that the N-terminal sequences of the 20-kDa and 60-kDa proteins were 100% identical, except for X, to those of clade D β-CA (GenBank accession number: KKP06884) and GPI (GenBank accession number: KKP03781) of *T. harzianum* strain T6776, respectively. Since some β-CA family enzymes exhibit COS-degrading activity, we regarded the 20-kDa protein as a putative COSase.

### Gene cloning and production of COSase in *E. coli*

**Fig. 2.**
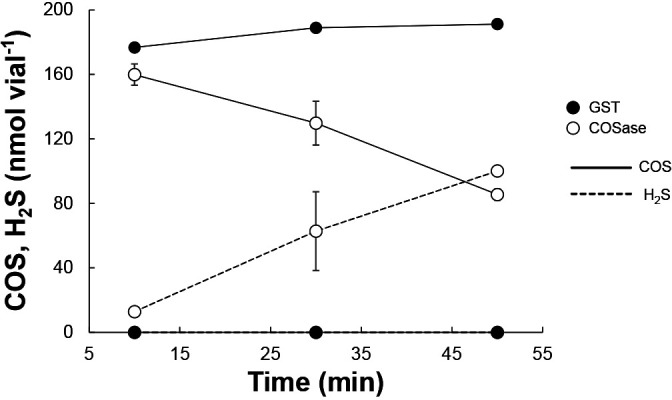
COS degradation and H_2_S production by purified recombinant COSase. Sampling of the headspace gas was started 10‍ ‍min after the addition of COS. Each point represents the average value of duplicate experiments. Error bars indicate maximum and minimum values.

The overall structure of the elucidated sequence regions of COSase and GPI is summarized in Fig. S3. Multiple alignments with similar nucleotide sequences to the reference strains are shown in Fig. S4. The elucidated coding sequences of COSase and GPI indicated that *T. harzianum* strain THIF08 contains genome regions with high similarities to CA (97%) and GPI (99.8%) of *T. harzianum* strain T6776. The cloning and expression of pGEX-COS and pGEX-GPI in *E. coli* revealed that only the recombinant protein from pGEX-COS caused a decrease in COS accompanied by the emission of H_2_S in the presence of 1,000‍ ‍ppmv of COS (190‍ ‍nmol in a 4.9-mL vial) (Fig. 2). The decrease observed in COS due to enzymatic degradation was balanced with the production of H_2_S, confirming the hydrolysis of COS. It was not possible to quantify the target protein used in the experiment shown in Fig. 2 because GST-fused proteins were not cleaved into the region of interest by the protease treatment, and, thus, a sufficient amount of enzyme was not obtained. Therefore, subsequent experiments on enzyme reactivities were performed using GST-fused COSase. In contrast, *E. coli* carrying pGEX-GPI did not exhibit COSase activity (data not shown). To the best of our knowledge, GPI has not been reported to degrade COS. Since GPI was co-purified with COSase through the purification step, GPI may have similar physicochemical properties to the COSase of *T. harzianum* strain THIF08. Alternatively, GPI may have bound to COSase through the purification step. Further research on GPI may provide another unknown function in the metabolism of this fungus. These results suggest that the COS-degrading ability and resulting H_2_S-producing ability of *T. harzianum* strain THIF08 are attributed to the COSase gene (*cos*).

### CA activity

The specific activity of WAU mg^–1^ of protein for bovine CA and COSase of *T. harzianum* strain THIF08 were 673.9 and 136.7, respectively. The time course of the pH decrease in the sigmoid curve of COSase was moderate and it took longer to reach pH 7.0 than bovine CA, which showed a steep sigmoid curve (Fig. S5). The specific activity of WAU mg^–1^ of protein for COSase was approximately one-fifth that of bovine CA. When experiments were performed with small amounts of COSase, such as 1‍ ‍μg (the amount equivalent to the enzyme of the region of interest), the pH change was consistent with that of the blank (Data not shown).

### Effects of CO_2_ on COS-degrading activity of COSase

**Fig. 3.**
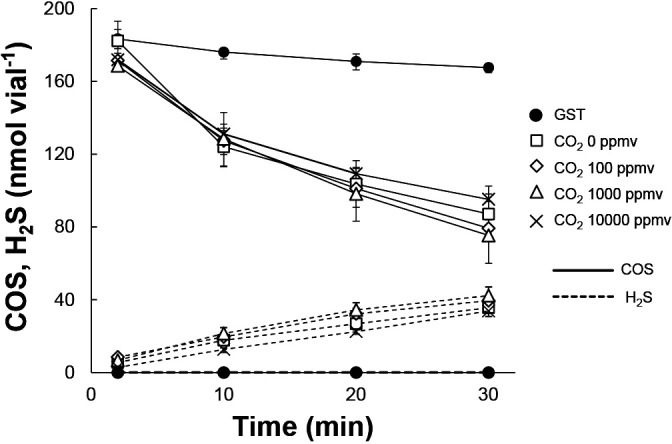
COS degradation and production of H_2_S by the purified protein of recombinant COSase under various concentrations of CO_2_. All symbols, except for the closed circle, contained COSase. Sampling of the headspace gas was started 2‍ ‍min after the addition of COS. Each point represents the average value of triplicate experiments. Error bars indicate maximum and minimum values.

Since CO_2_ is a substrate for CAs, the COS-degrading activity of COSase was examined in the presence of CO_2_ (100, 1,000, or 10,000 ppmv as the final concentration). Enzyme activity at 1,000 ppmv of COS (190‍ ‍nmol in a 4.9-mL vial) was 106‍ ‍nmol mg^–1^ min^–1^ in the first 10‍ ‍min and was unaffected by the presence of CO_2_ (Fig. 3).

### CS_2_ hydrolysis activity

CS_2_ hydrolysis activity was not detected at the CS_2_ concentration of 460‍ ‍μM previously used in the COSase assay for *T. thioparus* strain THI115 (Ogawa *et al.*, 2013). When a higher CS_2_ concentration, such as 80‍ ‍mM, was added, trace‍ ‍amounts of H_2_S were produced (approximately 3.8‍ ‍nmol‍ ‍mg^–1^‍ ‍min^–1^ over the first 10‍ ‍min) (Table S3). Therefore, the COSase of *T. harzianum* strain THIF08 may have weaker affinity for CS_2_ than the COSase and CS_2_ hydrolases already identified in prokaryotes.

### Multiple sequence alignment of COSase with other β-CA family enzymes

**Fig. 4.**
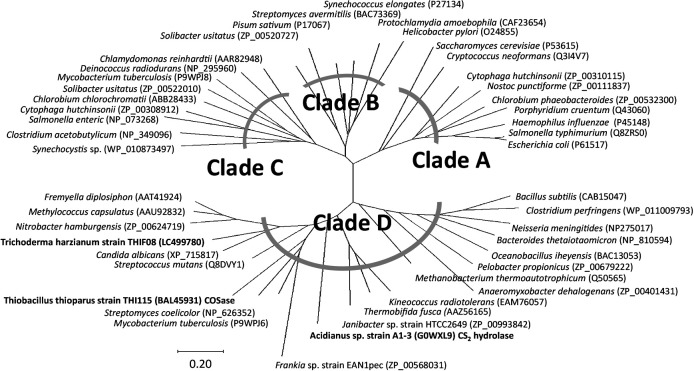
Phylogenetic tree of COSase, CS_2_ hydrolase, and β-CA family enzymes. A tree was constructed with MEGA X. β-CA family enzymes were selected from the Protein Data Bank and used the sequences from UniProt Knowledgebase (UniProtKB). β-CA family enzymes from the NCBI conserved domain database (CDD) were used to classify β-CAs into four clades, as described by Ogawa *et al.* (2013). The accession numbers of the β-CA family enzymes used here are indicated in parentheses. The organism name in bold indicates that the *in vitro* experiment on the organisms exhibited COS-degrading activity. Adapted with permission from (*J. Am. Chem. Soc.* 2013, **135**, 3818–3825). Copyright (2013) American Chemical Society.

**Fig. 5.**
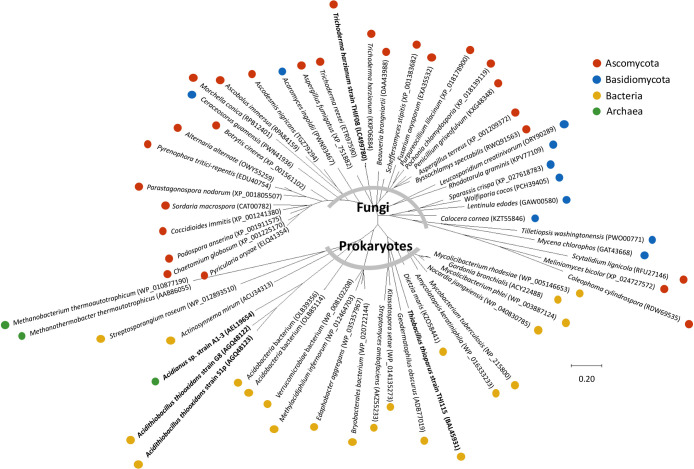
Phylogenetic tree of active sites of COSase, CS_2_ hydrolase, and clade D β-CAs from *Ascomycota*, *Basidiomycota*, *Bacteria*, and *Archaea*. The tree was constructed with MEGA X using the amino acid sequences of COSase, CS_2_ hydrolase, and clade D β-CAs. Detailed information on the protein dataset is provided in Table S4. The accession numbers of the protein sequences are indicated in parentheses. The organism name in bold indicates that an *in vitro* experiment on the organisms exhibited COSase activity.

The *cos* gene from *T. harzianum* strain THIF08 encoded a protein of 163 amino acid residues with a calculated molecular mass of ~18.05‍ ‍kDa, which is consistent with the molecular mass detected by the SDS-PAGE analysis (Fig. 1B). Due to the low mobility of the protein on Native-PAGE (~60‍ ‍kDa), strain THIF08 COSase was inferred to function as a trimer or tetramer. Comparisons of the amino acid sequence of *T. harzianum* strain THIF08 COSase with the previously known sequences of β-CA revealed that COSase belonged to clade D of β-CA family enzymes (Fig. 4) and exhibited approximately 31% amino acid sequence identity to COSase of *T. thioparus* strain THI115. The most closely related protein with COSase of strain THIF08 in the database was the CA of *T. harzianum* strain T6776, which has 97% amino acid sequence identity (Table S4). Comparisons of the amino acid sequence of strain THIF08 COSase to the active site of clade D β-CA family enzymes showed the distribution of similar amino acid sequences in fungi belonging to *Sordariomycetes*, *Eurotiomycetes*, *Dothideomycetes*, *Leotiomycetes*, *Pezizomycetes*, and *Saccharomycetes*. Although belonging to the same clade D cluster, the fungal clade D β-CA family enzyme was phylogenetically distinct from prokaryotic enzymes found in *Proteobacteria*, *Acidobacteria*, and *Actinobacteria* and in *Archaea*, such as *Crenarchaeota* and *Euryarchaeota* (Fig. 5 and Table S4). These results clearly show that clade D β-CA family enzymes are present not only in *T. harzianum* strain THIF08, but also in various other fungi, indicating a link between these enzymes and fungal COS uptake activity.

## Discussion

Increasing evidence suggests that a wide range of heterotrophic microorganisms, including bacteria (Kato *et al.*, 2008; Kusumi *et al.*, 2011) and fungi (Waksman, 1918; Wainwright, 1989; Li *et al.*, 2010; Xu *et al.*, 2018), play an important role in sulfur oxidation in soil; however, the biochemical pathways and enzymes involved remain unclear. The present study demonstrated the presence of the enzyme for the degradation of COS for the first time in fungi using *T. harzianum* strain THIF08. Comparisons of amino acid sequences from the database suggested the distribution of similar sequences in various *Ascomycota*; therefore, fungal COSase is a promising mediator of the biological transformation of atmospheric COS.

The COSase of *T. harzianum* strain THIF08 belongs to clade D in β-CA family enzymes, and amino acids at the predicted active site of COSase, including Cys36, His88, and Cys91, which are zinc ion coordinate residues (Rowlett, 2010), are also conserved in other fungal clade D β-CA family enzymes (Table S4). Similar active site amino acid sequences were also found in prokaryotic clade D β-CA family enzymes (Kato *et al.*, 2008; Smeulders *et al.*, 2011, 2013; Ogawa *et al.*, 2016; Kato *et al.*, 2020). CA is indispensable in many biological processes, such as CO_2_ fixation, respiration, and pH regulation, and the dominant CAs in bacteria and fungi belong to the β-CA family (Smith *et al.*, 1999; Smith and Ferry, 2000; Elleuche and Pöggeler, 2010). The β-CA family is considered to be evolutionarily ancient, existing before the divergence of the Archaea and Bacteria domains (Smith *et al.*, 1999). Since the CS_2_ hydrolases of *Acidianus* sp. strain A1-3 and *Acidithiobacillus thiooxidans* and the COSase of *T. thioparus* strain THI115 exhibited negligible or no CO_2_ hydration activities (Smeulders *et al.*, 2011, 2013; Ogawa *et al.*, 2013), enzymes in clade D may originally have stronger substrate affinity for COS than for CO_2_. In the present study, experiments were conducted using GST-fused COSase. A more accurate characterization of fungal COSase reactivity requires experiments using authentic COSase with the GST region removed.

The majority of filamentous ascomycetes encode multiple genes for β-CAs and also contain a gene for α-CA as a minor component (Elleuche and Pöggeler, 2009). Therefore, information on the catalytic reactivity of β-CA to COS is important for estimating the flux of COS between soil and the atmosphere. However, the kinetic information obtained from these enzymes is primarily limited to their reactivity to CO_2_. Bacterial and fungal COSase and related enzymes, such as archaeal and bacterial CS_2_ hydrolases, have all been shown to belong to clade D of β-CAs. Therefore, more detailed information is needed on the reactivity of clade D β-CAs to COS.

Genes of the clade D β-CA family with high similarity to that of strain THIF08 were also found in the genomes of *Ascomycota* and *Basidiomycota* (Table S4). *Ascomycota* are abundant in the upper layers of soils and considered to be the dominant saprotrophs (Lindahl *et al.*, 2007; Baldrian and Voriskova, 2013; Santalahti *et al.*, 2016), suggesting that the different relative abundance of *Ascomycota* may alter the COS exchange rate in soil. The metabarcoding analysis showed a positive correlation between *Ascomycota*-induced soil COS degradation and β-CA gene expression (Meredith *et al.*, 2018), and their role in driving COS exchange was predicted between soil and the atmosphere. The treatment of soil with the antifungal antibiotic nystatin affected soil COS uptake (Bunk *et al.*, 2017). Some fungal isolates belonging to *Sordariomycetes* (phylum *Ascomycota*) that were isolated from forest soil showed a strong ability (consumption rate per biomass) to degrade high concentrations of COS in the ppmv range (Masaki *et al.*, 2016). The relative abundance of fungi, particularly *Ascomycota* and *Basidiomycota*, correlated with soil COS flux and its CA transcript abundance, suggesting that fungi are important COS-degrading microeukaryotes in soil (Sauze *et al.*, 2017; Meredith *et al.*, 2018). While COS degradation by chemolithotrophic microorganisms is associated with energy production, the reason why chemoorganotrophic microorganisms exhibit strong COS-degrading activity in addition to the characteristics of the enzyme involved remain unclear.

Further studies on the characterization of the enzyme, including kinetic studies on COS and CO_2_, by assessing CA activity using other techniques, such as simultaneous variations in the CO^18^O isotopic exchange and COS fluxes (Jones *et al.*, 2017; Sauze *et al.*, 2017; Meredith *et al.*, 2018, Sauze *et al.*, 2018), are needed. A key entry point for explaining why clade D fungal β-CA has stronger affinity to COS than other CA family enzymes involves elucidating the biological function of COS degradation in fungi. Future research to compare the enzymatic characteristics of clade D β-CA family enzymes, particularly reactivity to CO_2_ and COS, is also required. The more precise estimation of COS uptake by microbes in nature will become possible if the mechanisms underlying COS degradation by microorganisms are clarified. These findings will contribute not only to our understanding of enzyme evolution, but also to mechanistically modeling and quantifying the role of soil microbial communities in the global COS budget (Ogée *et al.*, 2016).

## Citation

Masaki, Y., Iizuka, R., Kato, H., Kojima, Y., Ogawa, T., Yoshida, M., et al. (2021) Fungal Carbonyl Sulfide Hydrolase of *Trichoderma harzianum* Strain THIF08 and Its Relationship with Clade D β-Carbonic Anhydrases. *Microbes Environ ***36**: ME20058.

https://doi.org/10.1264/jsme2.ME20058

## Supplementary Material

Supplementary Material
