# Supplementary material for: Fungal Carbonyl Sulfide Hydrolase of Trichoderma harzianum Strain THIF08 and Its Relationship with Clade D β-Carbonic Anhydrases
Source: Microbes Environ. 2021 May 22;36(2):ME20058. doi: 10.1264/jsme2.ME20058 (PMC8209446; doi:10.1264/jsme2.ME20058)
Supplement: Supplementary file 1 — Supplementary Material [file 36_20058_s1.pdf]

Supplementary information

Fungal Carbonyl Sulfide Hydrolase of *Trichoderma harzianum* Strain THIF08 and Its Relationship with Clade D β-Carbonic Anhydrases

Yoshihito Masaki[1†#], Ryuka Iizuka[1#], Hiromi Kato[2], Yuka Kojima[3], Takahiro Ogawa[1,$], Makoto Yoshida[1], Yasuhiko Matsushita[4], and Yoko Katayama[1,5*]

[1]*Graduate School of Agriculture, Tokyo University of Agriculture and Technology, 3-5-8 Saiwai-cho, Fuchu, Tokyo 183-8509, Japan;* [2]*Graduate School of Life Sciences, Tohoku University, 2-1-1 Katahira, Sendai 980-8577, Japan;* [3]*Institute of Global Innovation, Tokyo University of Agriculture and Technology, 3-5-8 Saiwai-cho, Fuchu, Tokyo 183-8509, Japan;* [4]*Gene Research Center, Tokyo University of Agriculture and Technology, 3–5–8 Saiwai-cho, Fuchu, Tokyo 183–8509, Japan;* [5]*Independent Administrative Institution, Tokyo National Research Institute for Cultural Properties, 13-43 Ueno-Park, Taito-ku, Tokyo 110-8713, Japan*

# These authors contributed equally to this work.

†**Present address**: Chemical Management Center, National Institute of Technology and Evaluation, 2-49-10 Nishihara, Shibuya-ku, Tokyo 151-0066, Japan

$ **Present address**: Department of Biotechnology and Life Science, Tokyo University of Agriculture and Technology, 2–24–16 Naka-cho, Koganei, Tokyo 184–8588, Japan

Table S1. COS-degrading activity among various enzymes and associated enzyme kinetics*

Enzyme**	Kingdom	Organism name	k_{cat} (s^{-1})	K_m (μM)	k_{cat}/K_m ($s^{-1}M^{-1}$)	Reference
COSase	Bacteria	*Thiobacillus thioparus* strain THI115	58	60	$9.6×10^5$	Ogawa *et al*., 2013
CS_2 hydrolase	Archaea	*Acidianus* sp. strain A1-3	1800	22	$8.2×10^7$	Smeulders *et al*., 2011
CS_2 hydrolase	Bacteria	*Acidithiobacillus thiooxidans* strain S1p	810	74	$1.1×10^7$	Smeulders *et al*., 2013
CS_2 hydrolase	Bacteria	*Acidithiobacillus thiooxidans* strain G8	2200	14	$1.6×10^8$	Smeulders *et al*., 2013
β-CA	Plantae	*Pisum sativum*	93	39	$2.4×10^6$	Ogée *et al*., 2016
α-CA	Animalia	*Bos taurus*	41	$1.9×10^3$	$2.2×10^4$	Haritos *et al*., 2005
Nitrogenase	Bacteria	*Azotobacter vinelandii*	0.16	$3.1×10^3$	52	Seefeldt *et al*., 1995
CO dehydrogenase	Bacteria	*Rhodospirillus rubrum* ATCC11170	0.52	2.2	$2.4×10^5$	Ensign, 1995
RuBisCO	Bacteria	*Rhodospirillus rubrum*	6.3	$5.6×10^3$	$1.1×10^3$	Lorimer and Pierce, 1989
RuBisCO	Plantae	*Spinacia oleracea*	3.8	$1.8×10^3$	$2.2×10^3$	Lorimer and Pierce, 1989

* Modified from Ogawa *et al*., 2013. Adapted with permission from (J. Am. Chem. Soc. 2013, 135, 3818-3825). Copyright (2013) American Chemical Society.

** COSase, carbonyl sulfide hydrolase; CS_2 hydrolase, carbon disulfide hydrolase; CA, carbonic anhydrase; RuBisCO, ribulose-1,5-bisphosphate carboxylase/oxygenase.

Table S2. Oligonucleotides for cloning and sequencing

Name	Sequence (5' to 3')
ITS5_primer	GGAAGTAAAAGTCGTAACAAGG
ITS4_primer	TCCTCCGCTTATTGATATGC
TEF1_primer	GCCATCCTTGGAGATACCAGC
TEF728_primer	CATCGAGAAGTTCGAGAAGG
20kd_F01	GGAACCGTTTGCTCCAAGTGAGG
20kd_F02	CATTGCATTTCTCTTTTCACACAGC
20kd_R02	CTGAGATGAACTGGTGGTATTAATCTAC
20kd_R03	TTAAACATTAACCTTGTTAATCTTGCCCGTC
20kd_F06_EcoR	GCGAATTCATCGAAGGTCGTATGACCGTCGCCAGC
20kd_int1_R	AGTCAAGACAAAAACCTTTCGGCCGGGAGGCATAG
20kd_int1_F	CTATGCCTCCCGGCCGAAAGGTTTTTGTCTTGACT
20kd_int2_R	GCATACCGCAGTCGGTGTGATGGATAACAACGAC
20kd_int2_F	GTCGTTGTTATCCATCACACCGACTGCGGTATGC
20kd_R09_XhoI	GCGCCTCGAGTTAAACATCAACCTTGTTAATCTTG
60kd_F01	CTCTAATCAAGCATCTTCCCTC
60kd_F02	AACATGGCCCCCGCAAACACTC
60kd_F04	GACCCTCCACTTCGTCTCCAAC
60kd_F05	CGCATTATCGTGAGGCATTTCGAACC
60kd_F06	TCCGCCTGCTGCATCAATCCATCACC
60kd_F07	GTTCCTTGCTGGTGCTCACGCCATGG
60kd_R01	CAACCAGGCGAGTCTGGGCCTG
60kd_R02	CGTACCAGACGCCAAGCAGACC
60kd_R03	CGAGATCAGAGCCACCAATGCC
60kd_R04	GATGATGTTGGTCAGCTTCTTG
60kd_R05	ACCCTCCTTTTTCTCTGCATTTTCGG
60kd_R06	TTCTTTTCTTACACCGTATTTAACTG
60kd_R09	GCGCTGGATCCGTCAGAGGTAATGGC
GPI_F11_EcoRI	GCGAATTCATCGAAGGTCGTATGGCCCCCGCAAACACTCTTCCC
GPI_int1-R	TTACCATGACGGGGCCGAGATCAGAGCCACCAATG
GPI_int1-F	ATTGGTGGCTCTGATCTCGGCCCCGTCATGGTAAC
GPI_int2-R	GGAGGTATTGGTCGAAGGGAGCAACCAGGCGAGTC
GPI_int2-F	GACTCGCCTGGTTGCTCCCTTCGACCAATACCTCC
GPI_int3-R	CGCCAAACAGAATGGAGCCAGTGGTGTACTTGGCG
GPI_int3-F	CGCCAAGTACACCACTGGCTCCATTCTGTTTGGCG
GPI_R11_XhoI	GCGCCTCGAGTTAGATCTTGGAGAACGACTTG

Table S3. CS_2 hydrolysis activity of purified protein of recombinant GST-fused COSase measured by the production of H_2S*

Protein	CS_2**	
	460 μM	80 mM
GST-fused COSase	ND	28.8 ± 9.2 ppmv (3.8 ± 1.2 nmol mg^{-1} min^{-1})
GST	ND	ND

*Sampling of the headspace gas was in 10 min after the addition of CS_2. The amount of H_2S produced represents average value of triplicate experiments. Standard deviation of value is shown at right.

**ND means that concentrations of H_2S in the headspace gas were lower than 100 pptv of detection limit.

Table S4. Multiple sequence alignment of the active site of COSase, CS_2 hydrolase and clade D β-CA family enzymes from *Ascomycota*, *Basidiomycota*, *Bacteria* and *Archaea**

Phylum/Division	Class	Organism name**	Enzyme name***	Accession number	Used sequence			
Crenarchaeota	*Thermoprotei*	***Acidianus* sp. strain A1-3**	CS_2 hydrolase	AEL19654	Trp 31 - Gly 92	1	WVLTCMDERVHIEQSLGIQ--PDD------AHIYRNAGGIVTDDAIRSASLTTNFFGT---KEIIVVTHTDCG	62
Euryarchaeota	*Methanobacteria*	*Methanothermobacter thermautotrophicus* strain Delta H	CA	AAB86055	Cys 33 - Gly 96	1	CIITCMDSRLIDLLERALGIGRGD------AKVIKNAGNIVDDGVIRSAAVAIYALG---VNEIIIVGHTDCG	64
Euryarchaeota	*Methanobacteria*	*Methanothermobacter thermautotrophicus*	CA	WP_010877190	Cys 33 - Gly 96	1	CIITCMDSRLIDLLERALGIGRGD------AKVIKNAGNIVDDGVIRSAAVAIYALG---VNEIIIVGHTDCG	64
Acidobacteria	*Acidobacteria*	*Acidobacteria* bacterium 13 2 20CM 2 57 6	HP	OLB85114	Ala 32 - Gly 92	1	AVLTCMDTRLS-TRTLGLK--EGD------AHIIRNAGGIVTDDTLRSLVVSHHLLGT---EEFMVVNHTDCG	61
Acidobacteria	*Acidobacteria*	*Acidobacteria* bacterium 13 2 20CM 57 17	HP	OLB39356	Ala 32 - Gly 92	1	AVLTCMDTRLS-TRTLGLK--EGD------AHIIRNAGGIVTDDTLRSLVVSHHLLGT---EEFMVINHTDCG	61
Acidobacteria	*Acidobacteria*	*Bryobacterales* bacterium KBS 96	CA	WP_020722144	Ala 34 - Gly 95	1	AILTCMDARLDPAKFAGLS--EGD------AHVIRNAGGRATEDAIRSLVISYKLLGT---KEWFVIHHVDCG	62
Acidobacteria	*Acidobacteria*	*Edaphobacter aggregans*	CA	WP_035357967	Ala 34 - Gly 95	1	AILTCMDARLDPAKFAGLS--EGD------AHVIRNAGGRASDDAIRSLVISYKLLGT---REFFVIHHTNCG	62
Actinobacteria	*Actinobacteria*	*Actinosynnema mirum* DSM 43827	CA	ACU34313	Thr 32 - Gly 93	1	TILTCMDSRIRVFEIFGLK--QGE------AHVLRNAGGVVTDDMIRSLALSQRKLGT---REVLLVHHTNCG	62
Actinobacteria	*Actinobacteria*	*Amycolatopsis keratiniphila*	CA	WP_016333233	Ala 31 - Gly 92	1	AVLACMDARLNVYGALGLQ--EGE------AHVIRNAGGVVTEDEIRSLAISQRLLGT---REIILIHHTDCG	62
Actinobacteria	*Actinobacteria*	*Dietzia maris* DSM 43672	CA	KZO58441	Ala 32 - Gly 93	1	AVVACMDARLNPYGLLGLT--EGD------AHVIRNAGGVVTDDVLRSLTISQRLLGT---EEIVLIHHTDCG	62
Actinobacteria	*Actinobacteria*	*Geodermatophilus obscurus* DSM 43160	CA	ADB77019	Ala 31 - Gly 92	1	AVVACMDSRMPLFPMLGLE--VGD------AHVIRNAGGVITEDVIRSLTVSQHVLGT---REILLVHHTQCG	62
Actinobacteria	*Actinobacteria*	*Gordonia bronchialis* DSM 43247	CA	ACY22488	Ala 31 - Gly 92	1	AVVACMDARLDVYRILGLD--DGE------AHVIRNAGGVITDDEIRSLAISQRLLGT---TEIILIHHTDCG	62
Actinobacteria	*Actinobacteria*	*Kitasatospora*	CA	WP_014135273	Ala 41 - Gly 102	1	AVVACMDARLDLFAALGLE--LGD------AHIIRNAGGVVTDDAIRSLTISQRALGT---RTVVLIHHTGCG	62
Actinobacteria	*Actinobacteria*	*Mycolicibacterium phlei*	CA	WP_003887124	Ala 31 - Gly 92	1	AVVACMDARLDVYRILGLK--DGE------AHVIRNAGGVVTDDEIRSLAISQRLLGT---REIILIHHTDCG	62
Actinobacteria	*Actinobacteria*	*Mycobacterium tuberculosis* strain H37Rv	CA	NP_215800	Ala 31 - Gly 92	1	AIVACMDARLDVYRMLGIK--EGE------AHVIRNAGCVVTDDVIRSLAISQRLLGT---REIILLHHTDCG	62
Actinobacteria	*Actinobacteria*	*Mycolicibacterium rhodesiae*	CA	WP_005146653	Ala 31 - Gly 92	1	AVVACMDARLDVYRILGLK--DGE------AHVIRNAGGVITDDEIRSLAISQRLLGT---KEIILIHHTDCG	62
Actinobacteria	*Actinobacteria*	*Nocardia jiangxiensis*	CA	WP_040830785	Ala 31 - Gly 92	1	AVVACMDARLDVYRILGLG--EGE------AHVIRNAGGVVTDDEIRSLAISQRLLGT---TEIILIHHTDCG	62
Actinobacteria	*Actinobacteria*	*Streptomyces ambofaciens* ATCC23877	CA	AKZ55233	Ala 57 - Gly 118	1	AVVACMDARLDVTAALGLR--NGD------CHVIRNAGGAVTDDVIRSLTISQRALGT---RSVILVHHTACG	62
Actinobacteria	*Actinobacteria*	*Streptosporangium roseum*	CA	WP_012893510	Ala 32 - Gly 93	1	AVVTCMDSRIDPLGVFGLK--PGD------AKILRNAGARVTDDVLRTLVLAVYLLGV---NRVLVMPHTDCG	62
Proteobacteria	*Acidithiobacillia*	***Acidithiobacillus thiooxidans* strain G8**	CS_2 hydrolase	AGQ48122	Trp 32 - Gly 95	1	WVCACMDERLPVDDALGIR--GDR----GDAHVFRNAGGLITDDAIRSAMLTCNFFGT---EEIVIINHTECG	64
Proteobacteria	*Acidithiobacillia*	***Acidithiobacillus thiooxidans* strain S1p**	CS_2 hydrolase	AGQ48123	Trp 35 - Gly 102	1	WVLACMDERLPVDEALGIH--VDTPAGGGDAHCFRNAGGIVTDDAIRSAMLTCNFFGT---KEIVIVQHTQCG	68
Proteobacteria	*Betaproteobacteria*	***Thiobacillus thioparus* strain THI115**	COSase	BAL45931	Ala 40 - Gly 101	1	AVVACMDARLDVEDLLGLQ--TGE------AHIIRNAGGVINEDAIRCLIISHHLLNT---HEIILVHHTRCG	62
Verrucomicrobia	unclassified	*Methylacidiphilum infernorum*	CA	WP_012464703	Ala 34 - Gly 95	1	AILTCMDARLHPGKFAGLK--EGD------AHIIRNAGGRASDDAIRSLIISYKLLGT---KEWFVIHHTDCG	62
Verrucomicrobia	*Verrucomicrobiae*	*Verrucomicrobiae* bacterium strain DG 1235	CA	WP_008102208	Ala 34 - Gly 95	1	AILTCMDARLDPAKYAGLA--EGD------AHVIRNAGGRASDDAIRSLVISHKLLGT---NEWFVIHHTDCG	62
Ascomycota	*Dothideomycetes*	*Alternaria alternata* strain Z7	CA	OWY55259	Thr 34 - Gly 94	1	TIVTCMDARIDPTAAFGIP--LGA------AHVIRNAGG-CVKDAFRSIVISQQLLGT---REVILVKHTGCG	61
Ascomycota	*Dothideomycetes*	*Parastagonospora nodorum* strain SN15	HP	XP_001805507	Ala 38 - Gly 98	1	AVLTCMDARIDPTAAFGIP--LGA------AHVIRNAGA-SARDAFRSIVISQQLLGT---TEVLVVKHTGCG	61
Ascomycota	*Dothideomycetes*	*Pyrenophora tritici-repentis* strain Pt-1C-BFP	CA	EDU40754	Thr 34 - Gly 94	1	TIVTCMDARIDPTAAFGIP--LGA------AHVIRNAGA-SVKDAFRSIVISQQLLGT---REVMIVKHTCCG	61
Ascomycota	*Eurotiomycetes*	*Aspergillus fumigatus* strain Af293	CA	XP_751882	Ala 32 - Gly 92	1	AVVTCMDARIDVFSVLGLT--EGD------AHVIRNAGG-RASEALRSLIISQRLLGT---EEVVVIHHTDCG	61
Ascomycota	*Eurotiomycetes*	*Aspergillus terreus* NIH2624	HP	XP_001209372	Ala 32 - Gly 92	1	AIVACMDARLDVARALGLE--EGD------AHVIRNAGG-RVTDALRSLIISQQLLGT---REIIIVHHTDCG	61
Ascomycota	*Eurotiomycetes*	*Byssochlamys spectabilis* CBS 101075	CA	RWQ91563	Ala 32 - Gly 92	1	AVLACMDARLDPARALGLE--EGD------AHVIRNAGG-RVADAVRSLVISQQLLGT---REIVIVHHTDCG	61
Ascomycota	*Eurotiomycetes*	*Coccidioides immitis* strain RS	CD	XP_001241380	Leu 34 - Gly 94	1	LVLTCMDARIDPAAAFGID--LGD------AHVIRNAGA-NSRDALRSIIISQQLLNT---KEILLVKHTGCG	61
Ascomycota	*Eurotiomycetes*	*Penicillium griseofulvum* strain PG3	CA	KXG48348	Ala 32 - Gly 92	1	AVVACMDARLDPARVLGLE--EGD------AHVIRNAGG-RVSDALRSIIISQQLLGT---REIVIVHHTNCG	61
Ascomycota	*Leotiomycetes*	*Botrytis cinerea* strain B05.10	HP	XP_001561102	Ala 52 - Gly 112	1	AVVTCMDARIDPSAAFGIA--LGD------AHVIRNAGG-SARDALRSLVISQQLLGT---NEILLVKHTGCG	61
Ascomycota	*Leotiomycetes*	*Coleophoma cylindrospora* strain BP6252	HP	RDW69535	Val 42 - Gly 102	1	VIISCSDPRVIPEQFLDMN--LGEGA------VIRNAGG-RTMDAMRSILSLDAVGNL---GTIVVVHHTDCG	61
Ascomycota	*Leotiomycetes*	*Meliniomyces bicolor* strain E	HP	XP_024727572	Val 42 - Gly 102	1	VIISCADPRITPEHFLGLN--FGEAA------IIRNGGG-RTVDALRSILDLDSLGAT---GTVVVIHHTDCG	61
Ascomycota	*Leotiomycetes*	*Scytalidium lignicola* DSM 105466	HP	RFU27146	Val 43 - Gly 104	1	VVLSCSDPRLNPYQILGIDQ-TLKAT------MVRNAGG-RAFDAIRTLSVLQTIGAP---GTIVVMHHTDCG	62
Ascomycota	*Pezizomycetes*	*Ascobolus immersus* strain RN42	CA	RPA84159	Ala 41 - Gly 101	1	AIVTCMDARIHPSKAFGIT--LGD------AHIIRNAGA-SGVDALRSLVISQQLLGT---EEVFIVKHTDCG	61
Ascomycota	*Pezizomycetes*	*Ascodesmis nigricans* CBS 389.68	CA	TGZ79294	Ala 34 - Gly 94	1	AVVTCMDARLHPAKALGIN--LGD------AHIIRNAGG-SAQDALRSLTISQQLLGT---EVVVVVKHTDCG	61
Ascomycota	*Pezizomycetes*	*Morchella conica* strain CCBAS932	CA	RPB12401	Ala 36 - Ala 96	1	AVVTCMDTNIMPASALGFK--LGD------AHLIRNGGG-SAREALRSLVVSQQLFGT---KAILIIKHTDCA	61
Ascomycota	*Saccharomycetes*	*Scheffersomyces stipitis* CBS 6054	CD	XP_001383682	Ala 32 - Gly 92	1	AVVICMDARIDPAKALGLS--EGD------AHVIRNAGG-RASDALRSVIISQRLLGT---REIVVVHHTDCG	61
Ascomycota	*Sordariomycetes*	*Beauveria brongniartii* strain RCEF 3172	CA	OAA43988	Thr 32 - Gly 92	1	TVVTCMDARIDPAKILGLT--EGD------AHVIRNAGG-RAADSIRSLIISQQLLGT---REIVLIHHTDCG	61
Ascomycota	*Sordariomycetes*	*Chaetomium globosum* CBS 148.51	HP	XP_001225170	Leu 35 - Gly 95	1	LVLTCMDARIDPARAFGID--LGD------AHVIRNAGA-SAVDGLRSVVISQQLLGT---KEVILVKHTGCG	61
Ascomycota	*Sordariomycetes*	*Fusarium oxysporum* f. sp. *pisi* strain HDV247	CA	EXA35532	Ala 34 - Gly 94	1	AVIACMDARLDPAQVLGIE--LGS------AHVIRNAGG-RAADALRSVIISQQLLGT---REIVIVHHTDCG	61
Ascomycota	*Sordariomycetes*	*Pochonia chlamydosporia* strain 170	CA	XP_018139119	Ala 32 - Gly 92	1	AVVACMDARLDPAKVLGLS--EGD------AHVIRNAGG-RTIEALRSVVISQQLLGT---REIVILHHTDCG	61
Ascomycota	*Sordariomycetes*	*Podospora anserina* strain S mat+	UP	XP_001911575	Leu 33 - Gly 93	1	LVLTCMDARIDPARAFGIE--LGD------AHVIRNAGA-SAVDALRSIVISEQLLGT---NEIVLVKHTGCG	61
Ascomycota	*Sordariomycetes*	*Purpureocillium lilacinum* strain PLFJ-1	CA	XP_018178900	Ala 32 - Gly 92	1	AVVACMDARLDPAKILGLE--EGD------AHVIRNAGG-RTIEALRSIIISQQLLGT---REIAIVHHTDCG	61
Ascomycota	*Sordariomycetes*	*Pyricularia oryzae* strain Y34	CA	ELQ41354	Leu 34 - Gly 94	1	LVLTCMDARIDPAAAFGID--LGD------AHVIRNAGA-SARDGLRSVVISQLLLGT---REVLLLKHTGCG	61
Ascomycota	*Sordariomycetes*	*Sordaria macrospora*	CA	CAT00782	Leu 37 - Gly 97	1	LLLTCMDARIDPARAFGIE--LGD------AHVIRNAGA-STKDALRSIIISQQLLAT---EAIAIVKHTGCG	61
Ascomycota	*Sordariomycetes*	*Trichoderma harzianum* strain T6776	CA	KKP06884	Phe 32 - Gly 92	1	FVLTCMDARLDPAKVLGLE--EGH------AHVYRNAGG-RAAEALRSLIISQQALGT---EEVVVIHHTDCG	61
Ascomycota	*Sordariomycetes*	*Trichoderma reesei* strain RUT C-30	CA	ETR97590	Phe 32 - Gly 92	1	FVLTCMDARLDPAKFLGLE--EGH------AHVYRNAGG-RAAEALRSLIISQQALGT---REVVVIHHTDCG	61
Ascomycota	*Sordariomycetes*	***Trichoderma harzianum* strain THIF08**	COSase	LC499780	Phe 32 - Gly 92	1	FVLTCMDARLDPAKFLGLE--EGH------AHVYRNAGG-RAAEALRSLIISQQALGT---EEVVVIHHTDCG	61
Basidiomycota	*Agaricomycetes*	*Lentinula edodes* NBRC 111202	CA	GAW00580	Leu 32 - Gly 92	1	LIVTCMDARIDCYKSLGLE--LGE------AHIVRNAGG-SARDAFRSILISQRLLGT---REIAVFHHTNCG	61
Basidiomycota	*Agaricomycetes*	*Mycena chlorophos*	CA	GAT43668	Leu 30 - Gly 89	1	LVVTCMDPRIHPYEQFGLK--FGECG------IVRNGGG-STENALPSILIAQKFGG----HHIAVVHHTDCG	60
Basidiomycota	*Agaricomycetes*	*Sparassis crispa*	CA	XP_027618783	Ala 33 - Gly 93	1	AVVTCMDARLNVFGHLGIK--EGE------AHIIRNAGG-AAKDALRSIIISQRLLGT---REIAVFHHTACG	61
Basidiomycota	*Dacrymycetes*	*Calocera cornea* strain HHB12733	CA	KZT55846	Ile 31 - Gly 91	1	IVVTCMDARIDPAASLGIN--LGE------AHVIRNAGG-RATDALRSILISQRLLGT---SEIVLYHHTGCG	61
Basidiomycota	*Exobasidiomycetes*	*Acaromyces ingoldii* strain MCA 4198	CA	PWN93467	Ala 32 - Gly 92	1	AVLTCMDARILPEAALGIK--EGQ------AHVIRNAGG-RAPEALRSVIISQQLLGT---EEVVVVQHTDCG	61
Basidiomycota	*Exobasidiomycetes*	*Ceraceosorus guamensis* strain MCA 4658	CA	PWN41936	Thr 32 - Gly 92	1	TVLTCMDARIDPVAAFGIK--EGD------AHVIRNAGA-RAPDAVRSLVISQQLLGT---DTILVIGHSDCG	61
Basidiomycota	*Exobasidiomycetes*	*Tilletiopsis washingtonensis* strain MCA 4186	CA	PWO00771	Ile 62 - Gly 122	1	ILVTCMDGRILPEEAFGIK--AGTVN------VLRNAGG-RVAGAIPSILVSQVALGS---HEIIVVHHTDCG	61
Basidiomycota	*Hymenomycetes*	*Wolfiporia cocos* strain MD-104 SS10	CA	PCH39405	Ala 36 - Gly 96	1	AIVTCMDARLNVFAQLGLK--EGD------AHIIRNAGG-MARDAIRSIIISQRMLDT---REIAVFRHTLCG	61
Basidiomycota	*Microbotryomycetes*	*Leucosporidium creatinivorum* strain 62-1032	CA	ORY90289	Ala 53 - Gly 113	1	AIVGCMDARLDTSAATGLH--EGD------SHHIRNAGG-RAAEALRSLVISQELLGT---REIIVIHHTDCG	61
Basidiomycota	*Microbotryomycetes*	*Rhodotorula graminis* strain WP1	HP	KPV77109	Ala 62 - Gly 122	1	AVLCCMDARLDPNAMLGLE--VGD------AHIIRNGGG-RAADALRSLIGSQEALQT---REIIVIHHEDCG	61
Ascomycota	*Sordariomycetes*	*Sordaria macrospora*	CA	FM878639	Leu 41 - Gly 105	1	LWIGCSDSRCPETTILGMQ--PGD------VFVHRNIANIVSPTDINTTAVIEYAVAHLKVKHIVLCGHSACG	65

*Amino acid sequences used here were as follows: COSase of *Trichoderma harzianum* strain THIF08; clade D β-CA family enzyme of *Ascomycota* and *Basidiomycota* referred to Elleuche and Pöggeler (2010) and listed in the NCBI genome databases; the other clade D β-CA family enzyme referred to Ogawa *et al.* (2013, 2016). Sequence alignment was carried out on the active site that is homologous to the amino acid sequence of the active site of β-CA of *Sordaria macrospora* [CAS1 (FM878639), Leu41 to Gly105, Elleuche and Pöggeler, (2010)]. All sequences except for that of *S. macrospora* belong to the clade D β-CA family. The alignment was constructed using ClustalW with MEGA X. The residues that are identical in all sequences and identical in more than 50% are boxed in red and grey background, respectively. Closed stars indicate the zinc binding residues of β-CA. The accession number of each sequence is shown by NCBI.

** The name of organisms was based on the data from NCBI. The organism name in bold indicates that the *in vitro* experiment on the organisms exhibited the COSase activity. *Acidobacteria* bacterium 13 2 20CM 2 57 6 and 13 2 20CM 57 17; sequence data from soil metagenome.

*** The name of enzymes was based on the data from NCBI. CS_2 hydrolase, carbon disulfide hydrolase; CA, carbonic anhydrase; COSase, carbonyl sulfide hydrolase; CD, carbonate dehydratase; HP, hypothetical protein; UP, uncharacterized protein.

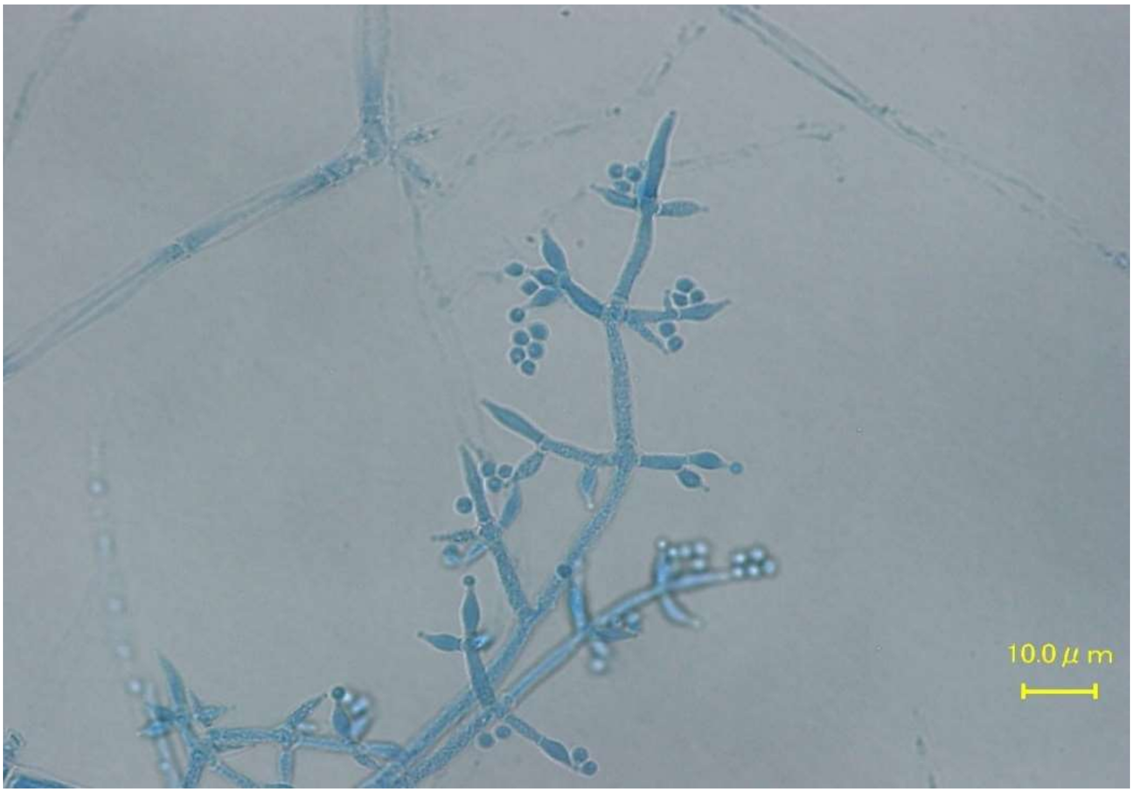

Fig. S1. Micrograph picture of *T. harzianum* strain THIF08 culture on PDA medium at 7 days.

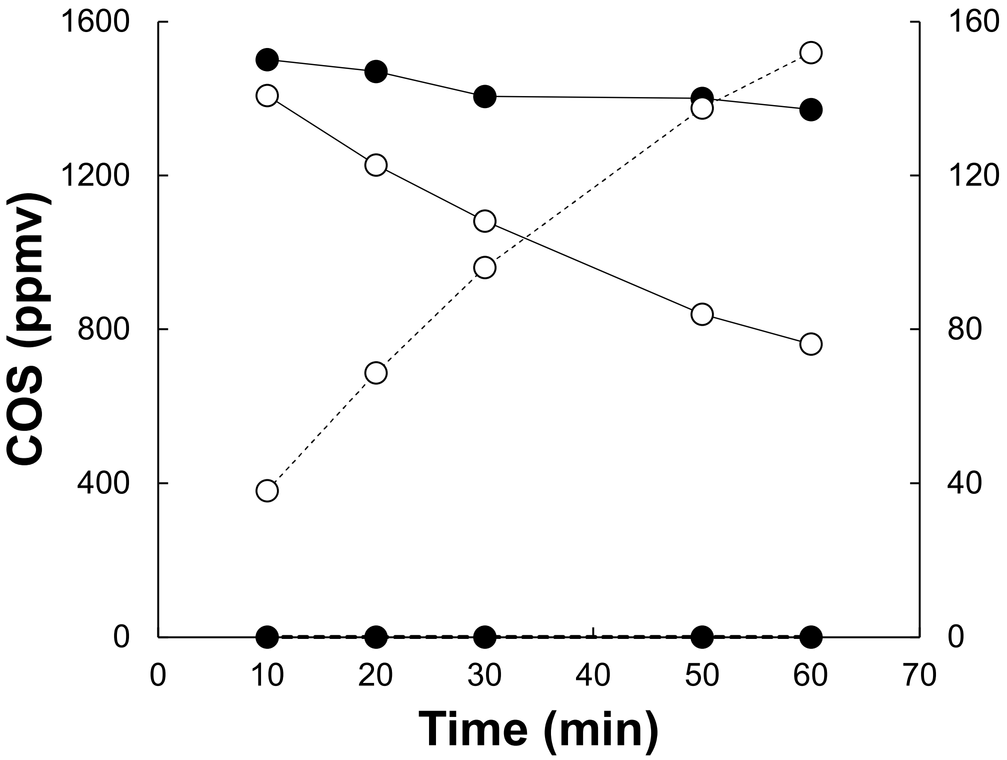

Fig. S2. COS degradation and production of H_2S by cell-free extract of *T. harzianum* strain THIF08. (Closed circle), cell-free extract after heat treatment; (open circle), cell-free extract. The amount of COS and H_2S are shown as solid line and dashed line, respectively. Sampling of the headspace gas was started 10 min after the addition of COS.

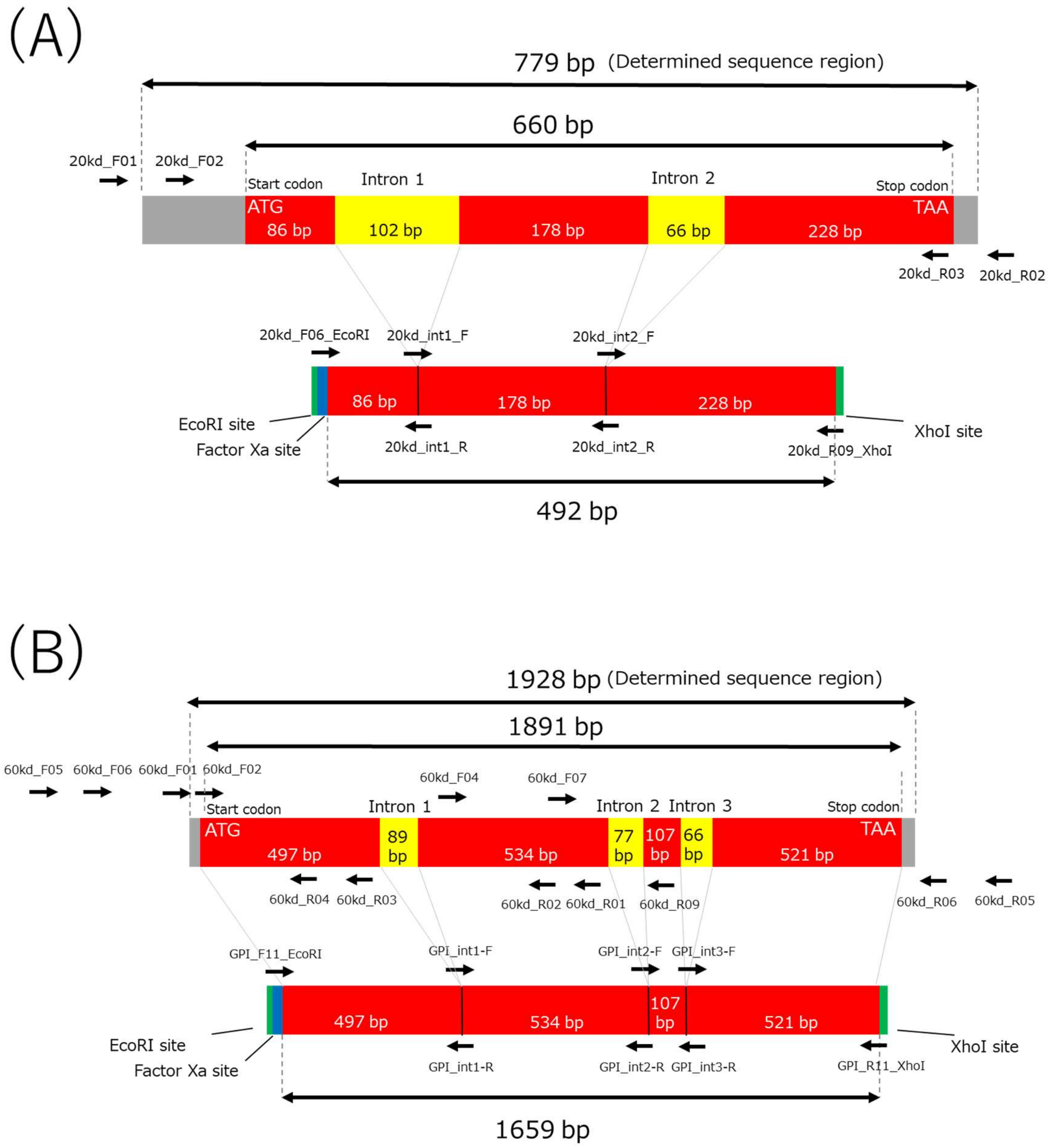

Fig. S3. Schematic summary of constructed genomic DNA fragments encoding (A) COSase and (B) GPI. (A) To amplify the DNA fragment encoding COSase, oligonucleotide primers for PCR were designed based on the genomic DNA sequence encoding CA (Joint Genome Institute [JGI] Protein ID: 784479) of *T. harzianum* strain TR274, in the JGI Genome Portal (https://genome.jgi.doe.gov) (Grigoriev *et al.*, 2012; Nordberg *et al.*, 2014). The primer sequences were as follows: 20kd_F01, 20kd_F02, 20kd_R02, and 20kd_R03 (Table S2). The total sequence size determined using primers was 779 bp. Sequence analysis of the amplified genomic DNA fragments revealed the presence of two putative introns (102 bp and 66 bp). To construct an expression plasmid, the putative exon regions (86 bp, 178 bp, and 228 bp) were amplified by using the two-step PCR method, and the fused protein-coding DNA fragment (492 bp) was cloned into

the expression vector pGEX-6P-1 (GE Healthcare Life Sciences) as follows. In the first PCR step, the three exon fragments (86 bp, 178 bp, and 228 bp) were amplified using the following primers: 20kd_F06_EcoRI and 20kd_int1_R, 20kd_int1_F and 20kd_int2_R, and 20kd_int2_F and 20kd_R09_XhoI (Table S2). In the second PCR step, the reaction mixture from the first step was used as a template, with the primers 20kd_F06_EcoRI and 20kd_R09_XhoI. The resultant DNA fragment containing the putative coding sequence of COSase was digested with the restriction enzymes EcoRI and XhoI, and cloned between the EcoRI and XhoI sites of pGEX-6P-1 to construct the expression plasmid pGEX-COS (5479 bp). The nucleotide sequence of the inserted DNA fragment was confirmed by sequencing. (B) For amplification of the genomic DNA encoding GPI, primers were designed based on the whole genome sequence of *T. harzianum* (DNA Data Bank of Japan [DDBJ] SRA data accession number: SRR976278). The primer sequences were as follows: 334 60kd_F05, 60kd_F06, 60kd_F01, 60kd_F02, 60kd_F04, 60kd_F07, 60kd_R05, 60kd_R06, 60kd_R09, 60kd_R01, 60kd_R02, 60kd_R03, and 60kd_R04 (Table S2). The total sequence size determined using primers was 1928 bp. Sequence analysis revealed the presence of three putative introns (89 bp, 77 bp and 66 bp). To construct the expression plasmid, the putative exon regions (497 bp, 534 bp, 107 bp and 521 bp) were amplified by using the two step-PCR method, and the fused protein-coding DNA fragment was cloned into pGEX-6P-1. In the first PCR step, the four exon fragments (497 bp, 534 bp, 107 bp and 521 bp) were amplified with the following primers: GPI_F11_EcoRI and GPI_int1_R, GPI_int1_F and GPI_int2_R, GPI_int2_F and GPI_int3_R, and GPI_int3_F and GPI_R11_XhoI (Table S2). In the second PCR step, the reaction mixture from the first PCR step was used as a template with the primers GPI_F11_EcoRI and GPI_R11_XhoI. The resultant DNA fragment containing the putative coding sequence of GPI was digested with EcoRI and XhoI, and then cloned between the EcoRI and XhoI sites of pGEX-6P-1 to construct the expression plasmid pGEX-GPI (6,646 bp). The nucleotide sequence of the inserted DNA fragment was confirmed by sequencing.

T. harzianum THIF08	1	TTCTCTTCATCTCCAAGCCATAT-CAAGACAGTCTTCAAGACATTGCATTTCTCTTTCCAT-CACCTTATTTAGCAACA-CACACTATCGAAAATGACCG	97
T. harzianum TR274	1	TTCTCTTCATCTACAAGCCATAT-CAAGACAGTCTTCAAGACATTGCATTTCTCTTTTCACACAGCTTATTTAGCAACA-CACACTATCGAAAATGACCG	98
T. harzianum T6776	1	TTCTCTTCACCTCCAAGCCATATTCAAGATAGTCTTCAAGACATTGCGTTTCTCTTTTCACACAGCTTATTTAGCAAGAGCACACTATCGAAAATGACCG	100
T. harzianum THIF08	98	TCGCCAGCGAGTTTGAAGTTGCCAACCAGCAATATGTTGCTACATTTGACAAGGCCGATCTGCCTATGCCTCCCGGCCGgtgcgtttcatctagctttca	197
T. harzianum TR274	99	TCGCCAGCGAGTTTGAAGTTGCCAACCAGCAATATGTTGCTACGTTTGACAAGGCCGATCTGCCTATGCCTCCCGGCCGgtgcgtttcacccagctttca	198
T. harzianum T6776	101	TCGCCAGCGAGTTTGAAGTTGCCAACCAGCAATATGTTGCTACATTCGACAAGGCCGATCTGCCTATGCCTCCTAGCCGgtgcgtttcatccagccttca	200
T. harzianum THIF08	198	ttattctctacagcaatccatcttttctacacagcaacttgaggattctcatgatttgctaatttgagatgagatacttagAAAGGTTTTTGTCTTGACT	297
T. harzianum TR274	199	ttattctctacagcagtccatcttttctacacagcaatttgagtattctcatgatttgctaatttgagatgagatatttagAAAGGTTTTTGTCTTGACC	298
T. harzianum T6776	201	tttttctctacacgaatccatcttttctaca---cgaattgagaattcccatgatttgctaatatgagatgagatatttagAAAGGTTTTTGTTCTGACT	297
T. harzianum THIF08	298	TGCATGGACGCTCGTCTGGACCCAGCCAAATTCCTTGGCCTTGAAGAGGGCCACGCCCACGTCTACCGCAATGCTGGAGGACGAGCTGCCGAGGCGCTGC	397
T. harzianum TR274	299	TGCATGGACGCTCGTCTGGACCCAGCCAAGTTCCTCGGCCTGGAGGAGGGCCACGCCCACGTCTACCGCAATGCCGGAGGACGAGCTGCCGAGGCGCTGC	398
T. harzianum T6776	298	TGCATGGACGCTCGTCTGGACCCAGCCAAGGTACTCGGCCTGGAAGAGGGCCACGCTCACGTCTACCGCAATGCCGGAGGACGAGCTGCCGAGGCGCTGC	397
T. harzianum THIF08	398	GCTCCTTGATCATTTCTCAGCAAGCTCTGGGAACCGAAGAAGTCGTTGTTATCCATCACgtatgaattgtctcccta-gaagctttgatgcacatagaat	496
T. harzianum TR274	399	GCTCCTTGATCATTTCTCAGCAAGCTCTGGGAACCGAAGAAGTTGTCGTTATCCACCACgtatgaattgtcccccca-gaagttttgatgcagatagaat	497
T. harzianum T6776	398	GCTCTTTGATCATTTCTCAGCAAGCTCTGGGAACCGAAGAAGTCGTCGTTATCCACCACgtatgaattgccccccccaaagctatacctatgatggaat	497
T. harzianum THIF08	497	tggttagctgacaagttattt-ataagACCGACTGCGGTATGCTTCTCATCCACGAGGAGGAGTTCCGCAACACTGTCAAGAAGAACACTGGCGAGGATG	595
T. harzianum TR274	498	tggctagctgacaaattattt-ataagACCGACTGCGGCATGCTTCTCATTCACGAGGAGGAGTTCCGCAACACTGTCAAGAAGAACACTGGAGAGGATG	596
T. harzianum T6776	498	tggttggttgacaaattatttataagACCGACTGCGGTATGCTTCTCATCCACGAAGAGGAGTTCCGCAACACTGTCAAAAAGAACACTGGCGAGGATG	597
T. harzianum THIF08	596	TCAGCCACATTGCATTCTTGACCATCCAGGACCTGCAGAAGAGCGTCAAGACTGATGTCGAGCTTTTGAGGAAGAATGCTGCGATCAAGAATGTGCCTAT	695
T. harzianum TR274	597	TCAGCCACGTTGCATTCTTGACCATCCAAGACCTGCAGAAGAGCGTCAAGACCGATGTCGAGCTTTTGAGGAAGAATGCTGCGATCAAGGATGTGCCTAT	696
T. harzianum T6776	598	TCAGCCACGTTGCATTCTTGACCATCAAGGATCTGCAGGAGAGCGTCAAGACTGATGTCGAGCTTCTGAGGAAGAATGCCGCGATCAAGAACGTGCCCAT	697
T. harzianum THIF08	696	TTCGGGCTACATCTTTGATGTCAAGACGGGCAAGATTAACAAGGTTGATGTTTAAGCGCATGTACGTCTAGAGGCAAATTATAT	779
T. harzianum TR274 v1.0	697	TTCGGGCTACATCTTTGATGTCAAGACGGGCAAGATTAACAAGGTTAATGTTTAAGCACATGTACGTCTAGAGGGAAAATATAT	780
T. harzianum T6776	698	TTCGGGCTACATCTTTGATGTCAAGACGGGCAAGATTAACAAGGTTGATGTTTAAGCGCATGTATGTGTAGA-------CATAT	774

Fig.S4 Multiple alignment of partial nucleotide sequences of *T. harzianum* strain TR274 and *T. harzianum* strainT6776 corresponding to sequence-determined region of *Trichoderma harzianum* strain THIF08 in this study. Nucleotides which are identical among three strains are indicated by asterisk. Nucleotide which is different from those of other two strains is shown in red. Nucleotides in putative COSase regions and intron regions are shown in bold upper case letter and lower case letter.

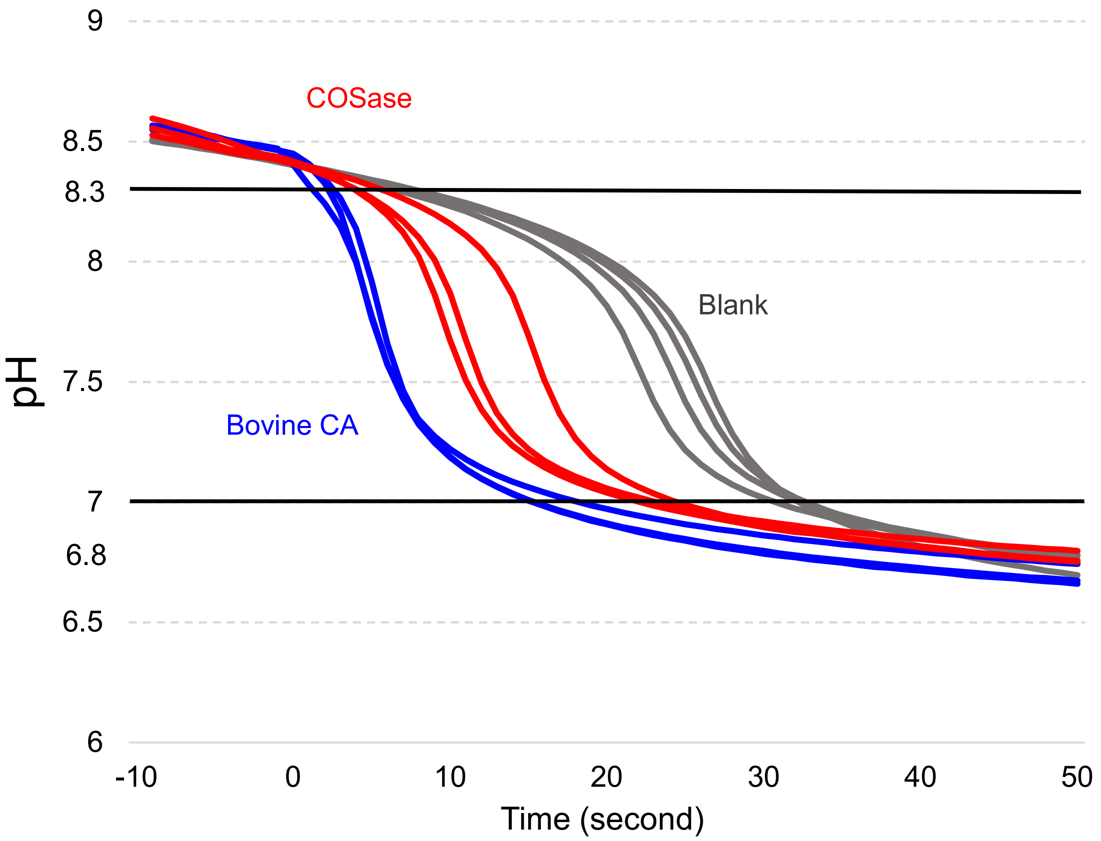

Fig. S5. Determination of the CA activity based on blanks and enzyme solutions. The time course of pH decrease from 8.3 to 7.0 was recorded. T_{Blank}, $T_{req(CA)}$ and $T_{req(COSase)}$ were 25.6, 15.3 and 20, respectively.

Supplementary references

Elleuche, S., and Pöggeler, S. (2010) Carbonic anhydrases in fungi. *Microbiol* **156**: 23–29.

Ensign, S. A. (1995) Reactivity of carbon monoxide dehydrogenase from *Rhodospirillum rubrum* with carbon dioxide, carbonyl sulfide, and carbon disulfide. *Biochemistery* **34**: 5372-5378.

Grigoriev, I. V., Nordberg, H., Shabalov, I., Aerts, A., Cantor, M., Goodstein, D., *et al.* (2012) The Genome portal of the department of energy joint genome institute. *Nucleic Acids Res* **40**: 26–32.

Haritos, V. S., and Dojchinov, G. (2005) Carbonic anhydrase metabolism is a key factor in the toxicity of CO_2 and COS but not CS_2 toward the flour beetle *Tribolium castaneum* [Coleoptera: Tenebrionidae]. *Comp Biochem Physiol C Toxicol Pharmacol* 140: 139-147.

Lorimer, G. H., and Pierce, J. (1989) Carbonyl sulfide: an alternate substrate for but not an activator of ribulose-1,5-bisphosphate carboxylase. *J Biol Chem* **264**:2764-2772.

Nordberg, H., Cantor, M., Dusheyko, S., Hua, S., Poliakov, A., Shabalov, I., *et al.* (2014) The genome portal of the department of energy joint genome institute: 2014 updates. *Nucleic Acids Res* **42**: 26–31.

Seefeldt, L. C., Rasche, M. E., and Ensign, S. A. (1995) Carbonyl sulfide and carbon dioxide as new substrates, and carbon disulfide as a new inhibitor, of nitrogenase. *Biochemistry* **34**: 5382-5389.

Smeulders, M. J., Barends, T. R. M., Pol, A., Scherer, A., Zandvoort, M. H., Udvarhelyi, A., *et al.* (2011) Evolution of a new enzyme for carbon disulphide conversion by an acidothermophilic archaeon. *Nature* **478**: 412-416.

Smeulders, M. J., Pol, A., Venselaar, H., Barends, T. R. M., Hermans, J., Jetten, M. S. M., and Op den Camp, H. J. M. (2013) Bacterial CS_2 hydrolases from *Acidithiobacillus thiooxidans* strains are homologous to the archaeal catenate CS_2 hydrolase. *J. Bacteriol Res* **195**: 4046-4056.

Ogawa, T., Noguchi, K., Saito, M., Nagahata, Y., Kato, H., *et al*. (2013) Carbonyl sulfide hydrolase from *Thiobacillus thioparus* strain THI115 is one of the β-carbonic anhydrase family enzymes. *J Am Chem Soc* **135**: 3818–3825.

Ogawa, T., Kato, H., Higashide, M., Nishimiya, M., and Katayama, Y. (2016) Degradation of carbonyl sulfide by Actinomycetes and detection of clade D of β-class carbonic anhydrase. *FEMS Microbiol Lett* **363**: fnw223.

Ogée, J., Sauze, J., Kesselmeier, J., Genty, B., VanDiest, H., Launois, T., and Wingate, L. (2016) A new mechanistic framework to predict OCS fluxes from soils. *Biogeosciences* **13**: 2221-2240.
